# Spatiotemporal and kinematic adjustments in master runners may be associated with the relative physiological effort during running

**DOI:** 10.3389/fspor.2023.1271502

**Published:** 2023-10-10

**Authors:** Parunchaya Jamkrajang, Sarit Suwanmana, Weerawat Limroongreungrat, Jasper Verheul

**Affiliations:** ^1^ College of Sports Science and Technology, Mahidol University, Nakhon Pathom, Thailand; ^2^ Cardiff School of Sport and Health Sciences, Cardiff Metropolitan University, Cardiff, United Kingdom

**Keywords:** aging, running biomechanics, performance, lower-Limb kinematics, spatiotemporal analysis

## Abstract

Master runners maintain a similar running economy to young runners, despite displaying biomechanical characteristics that are associated with a worse running economy. This apparent paradox may be explained by a greater physiological effort—i.e., percentage of maximal oxygen uptake (VO_2_-max)—that master runners perform at a given speed. Moreover, age-related responses to non-exhaustive sustained running are yet underexplored. The aims of this study were, therefore, to examine if biomechanical adjustments in master runners are physiological-effort dependent, and to explore the age-related biomechanical changes during a non-exhaustive sustained run. Young (23.9 ± 6; *n* = 12) and master (47.3 ± 6.9; *n* = 12) runners performed a sustained 30-minute treadmill run matched for relative physiological effort (70% VO_2_-max), while spatiotemporal and lower-limb kinematic characteristics were collected during the 1st and 30th minute. Group differences were observed in step/stride length, knee touch-down angle, and knee stiffness. However, both groups of runners had a similar step frequency, vertical center of mass oscillation, and knee range of motion. Age-related adjustment in these latter characteristics may thus not be an inevitable result of the aging process but rather a strategy to maintain running economy. The relative physiological effort of runners should, therefore, be considered when examining age-related adjustments in running biomechanics.

## Introduction

The benefits of distance running for attenuating age-related deterioration of musculoskeletal and cardiovascular health are increasingly recognized (1–5). Running participation at older ages can thus contribute to improved quality of life and help reduce the economic burden on healthcare systems. Accordingly, it is encouraging that the participation of master runners (i.e., > 35 years old) in long-distance running events has increased substantially over the last ∼40 years (6–8). Important motivational factors for running participation among master runners are competition and personal achievement (9, 10). Hence, understanding how aging affects running performance is essential for helping older individuals maintain running motivation.

The ability to sustain a high running speed for an extended period of time is fundamental for running performance, especially in long-distance events (races >5 km). It is well documented that long-distance running performance is closely connected to running economy (11–13)—i.e., the amount of oxygen consumed (VO_2_) at a given sub-maximal speed. The primary determinants of running economy are physiological and biomechanical in nature (14, 15). Physiological factors that can enhance running economy include a higher maximal oxygen uptake (VO_2_-max), an increased percentage of slow-twitch muscle fibers, and a higher stroke volume (15). From a biomechanical perspective, Moore (16) has pointed out several parameters that can positively affect running economy, such as running at a preferred stride length and frequency (i.e., spatiotemporal factors), less vertical oscillation, increased leg stiffness, and reduced lower-limb joint extension at take-off (i.e., lower-limb kinematic factors). Running performance, and changes thereof, are thus challenging to assess by considering physiological or biomechanical factors in isolation.

Declines in running performance appear to be an inevitable result of the aging process (17, 18). Age-related performance loss has been linked to several distinct physiological and biomechanical characteristics in master runners (19). Physiologically, aging introduces declines in, e.g., peak heart rate and VO_2_-max (20). Biomechanically, spatiotemporal and kinematic alterations, such as increased stride frequencies (21), and reduced lower-limb joint range of motion (22), have been shown to occur with increasing age. It is interesting to note that many biomechanical characteristics that are distinctive of master runners are also linked to a worse running economy (16), which in turn is an important determinant of distance running performance (11–13). Biomechanical alterations with age thus likely contribute to reductions in running economy and subsequently contribute to performance loss in master runners. Age-related changes in running kinematics and performance have indeed been linked previously in short- (23) and middle-distance (24) runners. However, trained master runners have also been shown to maintain a similar running economy at sub-maximal speeds compared to young runners, despite having distinct biomechanical characteristics (21, 25). Although running economy and performance are influenced by numerous factors, it is yet unclear how this apparent paradox can be explained.

Most studies that have investigated the biomechanical differences between master and young runners have matched the running speed for both groups. However, at a given sub-maximal running speed, master runners perform at a higher percentage of their VO_2_-max compared to young runners, despite having a comparable running economy (21, 26)—i.e., master runners perform a relatively greater physiological effort to maintain the same speed. It could, therefore, be that master runners adjust their running biomechanics in response to this greater physiological effort, which can contribute to reductions in performance despite maintaining a similar running economy. However, it is yet unclear if differences in running biomechanics are also present in master runners if the relative physiological effort, rather than the exact running speed, remains the same. Furthermore, changes in joint kinematics are known to occur after typical non-exhaustive running sessions—i.e., a duration of around 30–45 min, running at sub-maximal efforts such as 70%–85% of maximal heart rate or oxygen uptake (27, 28). Although there are indications that biomechanical adjustments after a bout of sustained running are comparable between master and young runners, evidence is still limited (29). The aim of this study was, therefore, twofold. First, to examine if differences in spatiotemporal and lower-limb kinematic characteristics are present in master runners if the overall physiological effort is matched with young runners. Second, to examine if changes in spatiotemporal and lower-limb kinematic characteristics during a typical non-exhaustive sustained run are similar between master and younger runners. We hypothesized (1) that age-related biomechanical adjustments depend on the relative physiological effort during running, and (2) that biomechanical responses to a sustained run are independent of age.

## Methods

### Participants

In total, 24 healthy recreational long-distance runners (i.e., competing in races >5 km) volunteered for this study (16 males and eight females). Participants were included if they met the age criteria for one of two age groups (Table 1)—either young runners (<35 years; *n* = 12) or master runners (>40 years; *n* = 12) (6). Only runners within the defined age brackets were included to ensure distinct age groups, and both groups were intentionally balanced for the number of male and female runners (Table 1). The sample size was determined based on a two-sided independent *t*-test (80% power, α = 0.05), considering a detectable difference of three degrees in knee range of motion, with a small to medium effect size (using G*Power 3.1, Universität Düsseldorf, Düsseldorf, Germany). All runners recruited for this study ran at least 25 kilometers per week in training and had completed at least one minimarathon race (10.55 km) in the last twelve months. Participants were excluded from this study if they had any current lower-limb injuries or previous surgery of the lower limbs in the past six months. All participants provided informed consent before participating in this study, in line with the ethical procedures, which were approved by the Mahidol University Central Institutional Review Board (reference number MU-CIRB 2019/132.0808).

**Table 1:** Comparison of young and master runners.

	Young runners	Master runners
Sex (#)	Males: eight	Males: eight
	Females: four	Females: four
Age (years)	23.9 ± 6	47.3 ± 6.9*
Mass (kg)	64.9 ± 7.1	61.4 ± 9.5
Height (cm)	172.1 ± 9.1	165.4 ± 8.6
Thigh length (cm)	38.8 ± 2	38.9 ± 3.1
Training volume (sessions/week)	3.58 ± 1.68	4.58 ± 1.24
VO_2_-max (ml/kg/min)	47.19 ± 7.28	41.01 ± 8.29
Running speed at 70% of VO_2_-max (km/hr)	7.92 ± 2.41	6.38 ± 1.49

Values are means ± standard deviations.

* = significant group difference (p < 0.05).

### Testing procedure and data analysis

The data collection consisted of two visits to Mahidol University's College of Sports Science and Technology biomechanics laboratory. During the first visit, participants performed a ten-minute familiarization and warmup run on the treadmill (Valiant 2 cpet, Lode, The Netherlands) at a self-selected running speed before performing a protocol to determine maximal oxygen uptake (VO_2_-max) (30–32). The VO_2_-max protocol consisted of an incremental run until exhaustion while oxygen uptake (VO_2_) was measured with a portable gas analyzer (Oxycon mobile, Jaeger, Germany). Running speed started at eight kilometers per hour and was increased by two kilometers per hour every 2 min. If a speed of 16 km per hour was reached, the incline of the treadmill was increased by 2% every 2 min. There were three termination criteria for the VO_2_-max protocol (33): (1) oxygen consumption during running reached a plateau with a plateau defined as two VO_2_ values over a 30-s period where the second value did not exceed the first value; (2) participants reached a respiratory exchange ratio of 1.15; (3) participants could not continue the protocol and asked to stop the test. Based on the results of the VO_2_-max protocol, each participant's running speed for the sustained run during the second laboratory visit was determined. A regression line was fitted to the VO_2­_ measurements across all running speeds. From this regression, each runner's speed at 70% of their VO_2_-max was determined.

During the second visit, all runners performed a sustained treadmill run during which their spatiotemporal and kinematic characteristics were assessed. Participants were asked to avoid high-intensity exercise and caffeine consumption in preparation for the second visit and wore their own running shoes in which they were most comfortable running. After a 10-minute warmup and familiarization on the treadmill, participants were instructed to perform a 30-minute sustained run on the treadmill (Walkerview performance 3.0, TecnoBody, Italy) at 70% of their VO_2_-max (27, 28). The combination of this duration and intensity was deemed non-exhaustive and reflective of a typical training run.

Three-dimensional kinematics were recorded using a motion capture system consisting of eight optoelectronic cameras (OptiTrack, NaturalPoint, USA), sampling at 100 Hz, and 44 retro-reflective markers (16 mm diameter) were attached to the participant's body following the lower limb and trunk model (34). Prior to each 30-min treadmill run, a static trial was captured. Throughout the run, kinematic data for five strides were collected during the 1st and 30th minute. Visual 3D (version 6.0, C-motion, Germantown, USA) was then used to process the marker trajectories and analyze kinematics. Marker trajectories were gap-filled and filtered at 9 Hz using a fourth order Butterworth lowpass filter. An eight-segment musculoskeletal model was then built using the static trial, from which segment and joint kinematics during running were derived. Inverse kinematic constraints were applied to each of the lower limb joints. Several discrete kinematic variables were then determined to assess running kinematics for each runner.

### Spatiotemporal and kinematic characteristics

For each of the five strides that were collected at both time points (i.e., 1st and 30th minute), the moments of touch-down and take-off were identified using the minimal vertical velocity of the pelvis center of mass (35) and peak knee extension (36) respectively. Touch-down and take-off were used to determine spatiotemporal running characteristics—i.e., stride and step length, and step frequency. Kinematic characteristics included the joint angles of the hip, knee, and ankle, and the whole-body center of mass (CoM) position (which was normalized to each participant's height) at touch-down and take-off. In addition, the range of motion of each joint and the CoM during the stance phase was determined. Since bilateral kinematic asymmetries are common, can be substantial, and can affect group comparisons (37–40), leg-specific comparisons were made between the master and young runners.

**Figure 1:**
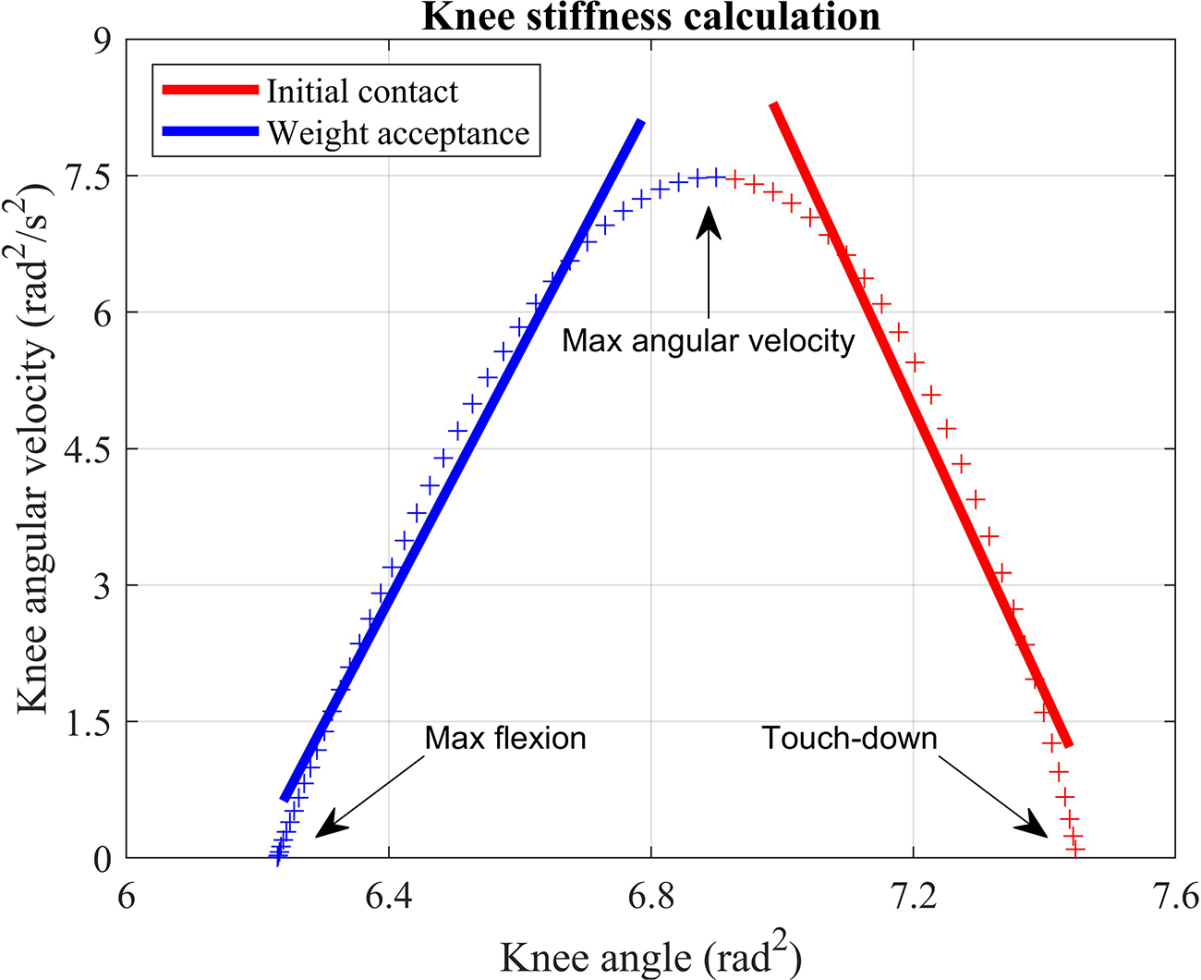
A representative example trial of the kinematics-based calculation of the knee stiffness during the initial contact (red) and weight acceptance (blue) phase of landing.

Stiffness of the knee joint was calculated using a previously described and validated kinematics-based method (Figure 1) (28, 41–43). Briefly, knee stiffness values K_knee_ were determined for the initial contact phase (from touch-down to the peak knee angular velocity) and the weight acceptance phase (from peak knee angular velocity to maximal knee flexion) according to:

$$K_{\mathrm{knee}}=\frac{I\cdot \frac{\Delta \omega^{2}}{\Delta \theta^{2}}}{\mathrm{ROM}}$$

in which *I* is the participant's mass multiplied by the length of the thigh squared ($m\;\;\cdot \;l_{thigh}^{2}$), ω is the knee angular velocity in rad⋅s^−1^, θ is the knee angle in radians, and ROM is the range of motion of the knee in degrees, for either the initial contact or weight acceptance phase of landing. The thigh length was measured as the distance between the greater trochanter and lateral femoral epicondyle (Table 1). $\frac{\Delta \omega^{2}}{\Delta \theta^{2}}$ was determined by up-sampling the knee angle data between touch-down and maximal knee flexion to 500 Hz and fitting a line to the data points between 20%–80% of each phase. Further details and examples can be found in Dutto and Braun (28), Verheul et al. (42), and Zhang and Lake (43).

### Statistical analysis

Since bilateral kinematic asymmetries are common and can be substantial (37, 38, 40), we performed leg-specific statistical analyses for each kinematic variable. SPSS (version 18.0, IBM, Chicago, IL, USA) was used to perform statistical analyses. A Shapiro–Wilk test was used to determine the normal distribution of discrete data. Group characteristics (Table 1) were compared using *t*-tests, and effect sizes were assessed using Cohen's d as small (*d* = 0.2), medium (*d* = 0.5), or large (*d* = 0.8). A two-way repeated measures ANOVA was used to evaluate the effect of age (i.e., master vs. young runners) and running time (1st and 30th minutes). Holm *Post-hoc* tests were used to determine the locations of significant effects. All data are reported as mean ± standard deviation (SD). The level of statistical significance was set at *p* < 0.05. To enhance result comparisons across studies (44), effect sizes were determined by calculating the partial eta squared ($\eta_{p}^{2}$). Effect sizes were evaluated as either small ($\eta_{p}^{2}$ = 0.01), medium ($\eta_{p}^{2}$ = 0.06), or large ($\eta_{p}^{2}$ = 0.14) effects (45).

## Results

Young runners had a higher (*p* = 0.07, *d* = 0.8) VO_2_-max compared to the master runners (Table 1). Accordingly, the running speed at 70% of VO_2_-max was reduced (*p* = 0.07, *d* = 0.8) in the master group, and the young runners completed a greater distance (3.96 ± 1.2 km) during the 30-minute treadmill run than the master runners (3.19 ± 0.74 km). Although these differences were not significant, the effect sizes were large.

### Spatiotemporal characteristics

**Figure 2:**
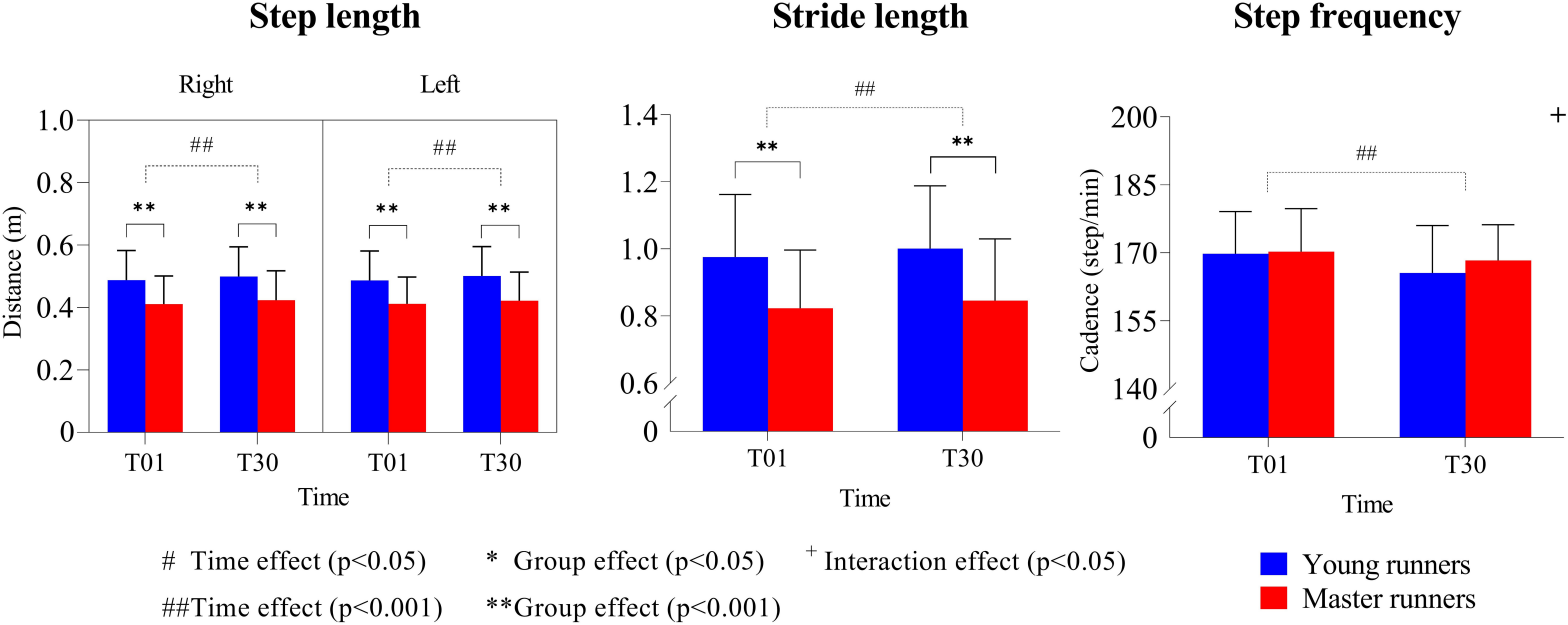
Means and standard deviations for step length (left and right), stride length, and step frequency during running. T01 = 1st minute; T30 = 30th minute.

**Figure 3:**
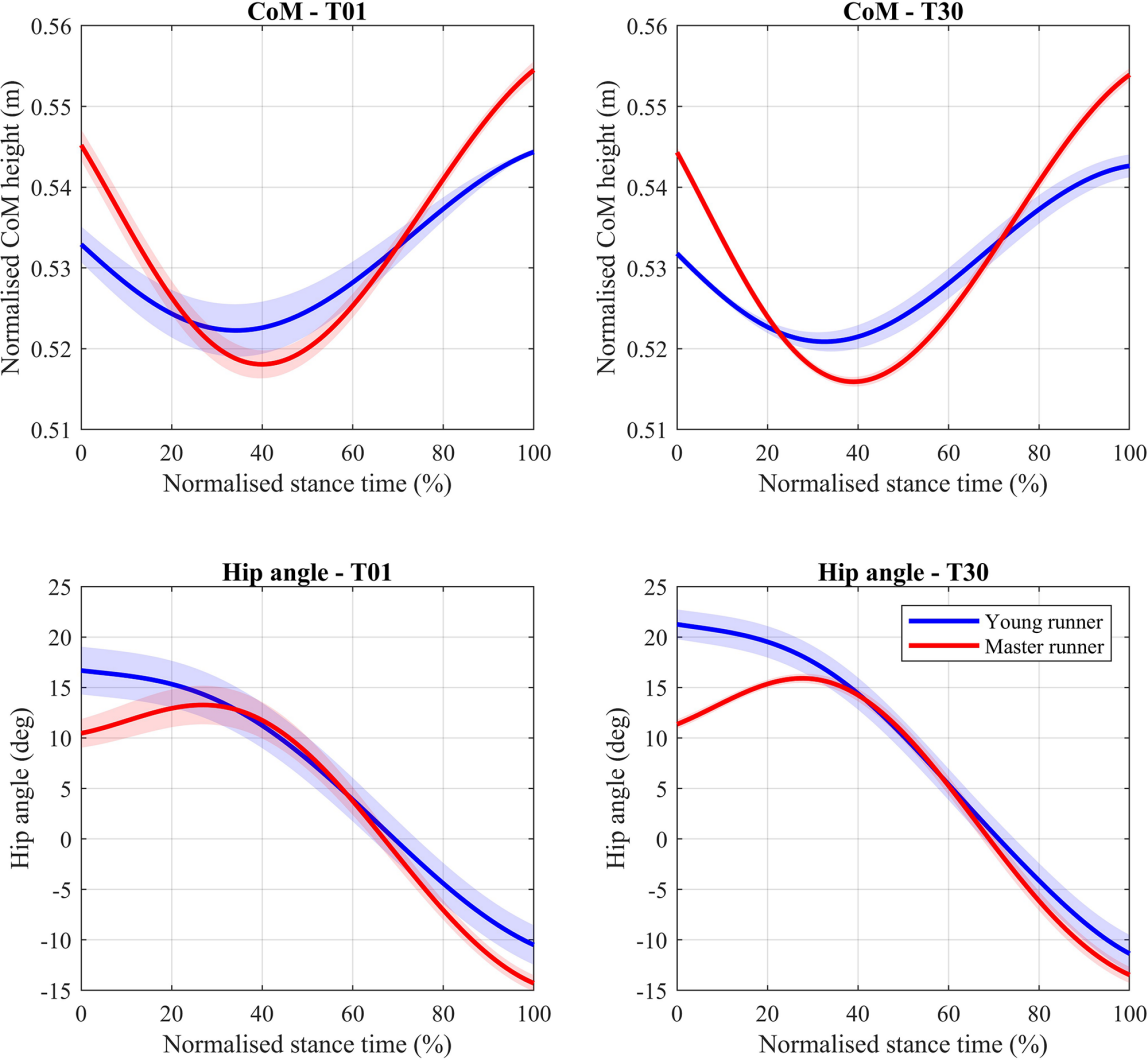
Centre of mass (CoM; top row) and hip angle (bottom row) trajectories during the stance phase of running for one representative young (blue) and master (red) runner. Curves represent means and standard deviations (shaded areas) over five ground contacts. Data are shown for the start (T01) and end (T30) of the sustained 30-minute run.

Step lengths were significantly longer by 16% (*p* < 0.001) for the young compared to the master group, for both legs with a large effect size (right $\eta_{p}^{2}$ = 0.16; left $\eta_{p}^{2}$ = 0.15) (Figure 2). Moreover, in both groups the step lengths significantly increased (*p* < 0.001; $\eta_{p}^{2}$ = 0.08–0.1) after the 30-minute sustained run. Consequently, stride lengths were also longer for the young runners (16%; *p* < 0.001; $\eta_{p}^{2}$ = 0.16) and increased by 3% over time (*p* < 0.001; $\eta_{p}^{2}$ = 0.12) (Figure 2). Step frequency, however, was similar for both groups and significantly decreased (*p* < 0.001; $\eta_{p}^{2}$ = 0.21) after 30 min of running (Figure 3), with a small interaction between age and running time (*p* < 0.05; $\eta_{p}^{2}$ = 0.04).

### Kinematic characteristics

**Figure 4:**
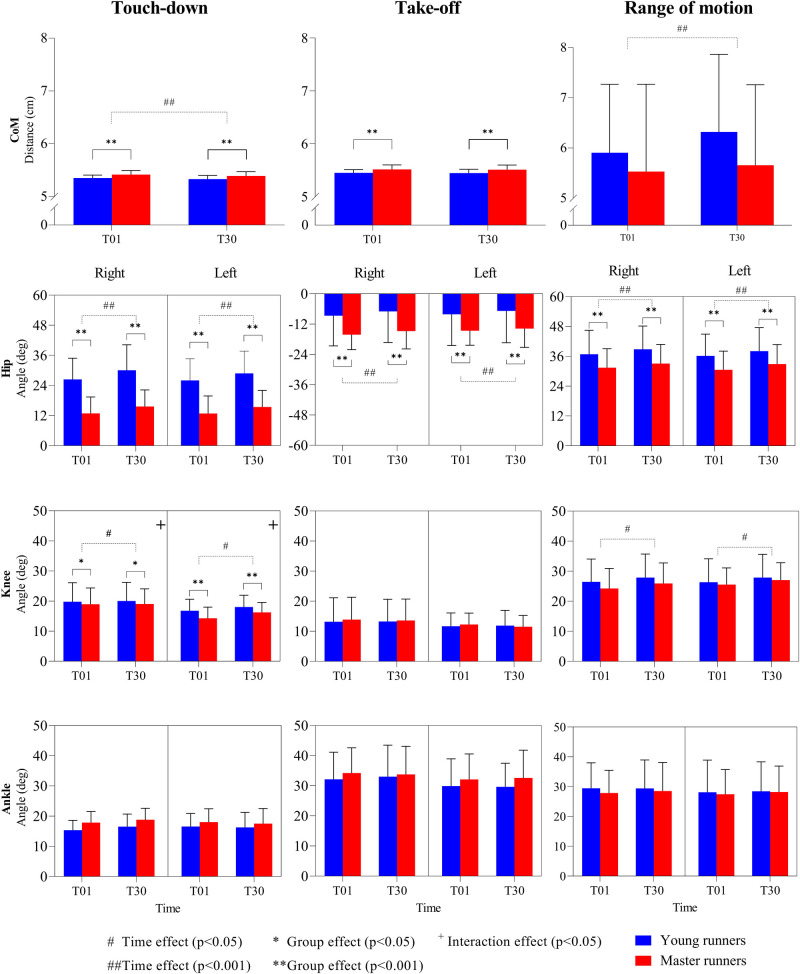
Means and standard deviations of the center of mass position (CoM) and hip, knee, and ankle joint angles (left and right), at touch-down and take-off, and the range of motion during the stance phase of running. Positive-negative angles indicate flexion-extension (hip and knee), and plantarflexion- dorsiflexion (ankle). T01 = 1st minute; T30 = 30th minute.

The normalized vertical position of the CoM was significantly higher at touch-down in the master runners compared to the young group (1%; *p* < 0.001; $\eta_{p}^{2}$  = 0.18), whereas running time significantly lowered the position of the CoM at touch-down in both groups (1%; *p* < 0.001; $\eta_{p}^{2}$ = 0.32) (Figure 4—top row). At take-off, however, the vertical CoM position was significantly higher in the master runners compared to the young runners (1%; *p* < 0.001; $\eta_{p}^{2}$ = 0.16), but the sustained run did not affect this. Furthermore, age did not affect the range of motion of the CoM, but running time did by 5% (*p* < 0.001; $\eta_{p}^{2}$ = 0.15).

The angles of the hip and knee joints at touch-down were significantly larger (48%–50% and 11%–13% respectively; p < 0.05) for the young runners compared to the master group, both for the right (hip $\eta_{p}^{2}$ = 0.45; knee $\eta_{p}^{2}$ = 0.06) and left (hip $\eta_{p}^{2}$ = 0.44; knee $\eta_{p}^{2}$  = 0.16) leg (Figure 4—left column). The sustained run caused significant increases in the hip (14%–17%; *p* < 0.001; $\eta_{p}^{2}$ = 0.41–0.45) and knee (1%–4%; *p* < 0.05; $\eta_{p}^{2}$ = 0.08–0.09) joint angles for both legs at touch-down with medium to large effect sizes. There was also small to medium interaction between age and running time for knee touch-down angle for both legs (*p* < 0.05; $\eta_{p}^{2}$  = 0.04–0.07). The ankle joint angle at touch-down, however, was neither affected by age nor running time.

Age and 30 min of sustained running both significantly (*p* < 0.001) affected the hip joint angle at take-off (Figure 4—middle column). For both legs, there were medium to large 90%–98% increases in hip angle with age ($\eta_{p}^{2}$ = 0.11–0.14), but a large 9%–12% decrease with running time ($\eta_{p}^{2}$ = 0.14–0.2). However, the knee and ankle joint angles at take-off were unaffected by age or running time.

The range of motion of the hip during the stance phase was significantly greater in the young runners compared to the master runners (15%; *p* < 0.001; = 0.1) for both the right and left leg (Figure 4—right column). Likewise, sustained running had a large increasing effect (5%–6%; *p* < 0.001; $\eta_{p}^{2}$ = 0.23–0.35) on the hip range of motion in both age groups. Although age did not affect the range of motion of the knee or ankle joints, the knee range of motion was significantly increased after the sustained run in both legs (3%–6%; *p* < 0.05; $\eta_{p}^{2}$ = 0.06–0.12).

### Knee joint stiffness

**Figure 5:**
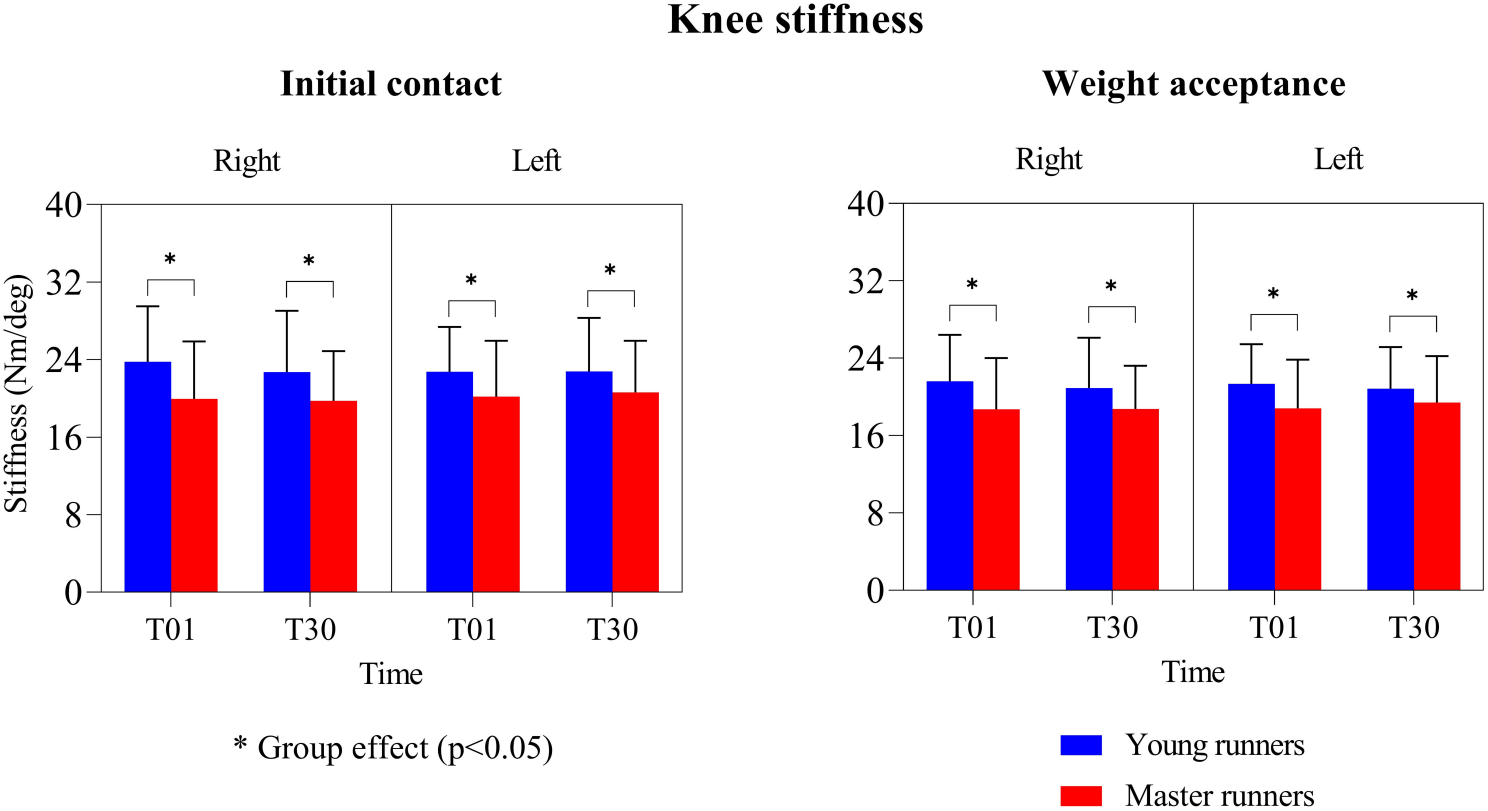
Means and standard deviations of left and right knee stiffness during the initial impact and weight acceptance phase. T01 = 1st minute; T30 = 30th minute.

A medium significant effect of age (*p* < 0.02; $\eta_{p}^{2}$ = 0.05–0.09) on knee stiffness (Figure 5) was found. In both legs, a 9%–15% higher knee stiffness was found for the young runners compared to the master runners during both the initial impact and weight acceptance phases of landing. However, knee stiffness was not affected by running time.

## Discussion

Well-trained master runners have been found to have a similar running economy compared to young runners, despite showing altered biomechanics that are related to a worse running economy. The results of this study show that when physiological effort is matched, master runners run with a different step length, knee touch-down angle, and knee stiffness, compared to younger runners. However, step frequency, vertical center of mass oscillation, and knee range of motion were similar between both groups. These findings indicate that age-related adjustment in step frequency, vertical center of mass oscillation, and knee range of motion may not be an inevitable result of the aging process but rather a strategy to maintain running economy.

An essential component of running performance is speed, which is determined by the combination of step length and frequency. Runners are known to self-optimize these parameters to get close to their economical optimum for a given speed (46). We found that both the step and stride length were significantly reduced in the master runners compared to the young runners, while step frequency was the same in both groups. Several others have reported similar reductions in step and stride length for master runners, but these were always accompanied by increases in step frequency to maintain speed (21, 22, 47, 48). In this study, however, each runner's speed was matched to be at 70% of their VO_2_-max and hence, running speeds varied across runners (Table 1). Our results thus show that master runners who run at the same physiological effort adjust (i.e., lower) their step length but maintain the same step frequency, which leads to the observed reductions in speed. In other words, running performance loss in master runners is primarily associated with changes in step length but not frequency. In line with our first hypothesis, step frequency adjustments are thus likely to be dependent on physiological effort and not age.

An increased range of motion of the body's CoM during the stance phase of running (or vertical oscillation) is associated with a worse running economy (16, 49). We did not find any differences in vertical oscillation between both groups of runners. In contrast, Karamanidis and Arampatzis (48) found that master runners run with less vertical oscillation than young runners, which should thus contribute to a better running economy. Since that study used the same speed for all runners, and the master group ran at a greater (perceived) physiological effort (i.e., higher percentage of VO_2_-max), it is possible that the master runners adjusted their vertical oscillation to a more economical pattern to maintain running economy. Since we matched each runner's running speed to be at the same physiological effort, together, these results provide evidence that master runners adjust their vertical oscillation depending on the relative physiological effort, which supports our first hypothesis.

Running with more knee flexion at touch-down and a larger knee joint range of motion has been linked to a higher metabolic demand—i.e., a worse running economy (50–52). Previous studies have found older runners to run with a more flexed knee at touch-down and a smaller knee joint range of motion (22, 25, 47), which would respectively contribute to a worse and better running economy, and thus a negligible net change. In our study, however, master runners only ran with a less flexed knee at touch-down but had a similar knee range of motion to young runners, which together would contribute to a better running economy. Hence, master runners adjust multiple knee characteristics (i.e., touch-down angle and range of motion) when the relative physiological effort to perform the same running speed increases with age, to help maintain a similar running economy (21). Since only the knee touch-down angle was different from the young runners in our study, adjustments of the touch-down angle of the knee joint are likely to be independent of effort and primarily age-related. Adjustments of the knee range of motion, however, are likely to be physiological-effort dependent, which further supports our first hypothesis.

Good evidence exists for a positive correlation between running economy and the amount of flexion of the lower-limb joints at take-off (53, 54). Although we did not observe any group differences for the take-off angle of the ankle and knee joints, master runners ran with a significantly more extended hip joint at take-off (together with a lower hip range of motions and less hip flexion at touch-down). This can be a contributing factor to a less economic running style and performance loss. Interestingly, not many previous studies have investigated the kinematics of the hip joint during running in older populations. Future work should, therefore, further examine the relationship between hip flexion at take-off and running economy, and possibly consequent performance loss, in master runners.

An increased stiffness of the knee joint is related to better running economy and performance (55, 56). We found the knee to be 7%–19% stiffer in young compared to master runners, both during the initial contact phase and the weight acceptance phase, regardless of the effort-matched design in this study. This decrease in knee stiffness with age is in line with previous findings of ∼10%–20% stiffness declines in the knee and ankle joints (57), and leg (21) in older runners. Stiffness reductions have primarily been linked to the loss of muscle strength and neuromuscular function due to aging, both of which may be slowed down by running training (21, 58, 59). It has indeed been shown that (young) runners with a higher training volume can more efficiently coordinate the thigh muscles to regulate knee stiffness during landing (42), whereas older runners with a consistently high training volume can retain their stiffness regulation capacity (58, 60). These findings support the notion that sufficiently high running volume might positively contribute to the maintenance of appropriate leg and joint stiffness in master runners and can help attenuate economy and performance loss with aging.

Changes in joint kinematics have been shown to occur over the course of a sustained run (27, 29, 61). Our findings further support the notion that non-exhaustive sustained running affects lower-limb kinematics. We found that 30 min of running at 70% of VO_2_-max increased the step and stride length, decreased the step frequency, increased the hip and knee joint angles at touch-down, lowered the CoM position at touch-down, decreased the hip angle at take-off, and increased the range of motion of CoM, hip, and knee. However, only for the step frequency and the touch-down angle of the knee did we observe a significant interaction between age and running time, but the effect sizes were small to medium. Together, these findings are largely in line with a previous work (29) that did not find sustained running to affect the running biomechanics of master and young runners differently and supports our second hypothesis that biomechanical responses to a sustained run are largely independent of age.

Bilateral kinematic asymmetries are common and can be substantial, although clear evidence of the detrimental effects of asymmetries on running economy and performance is lacking (37, 38, 40). However, given the high asymmetry prevalence, either focusing on a single leg or combining both legs together in the same analyses can introduce biases in kinematic comparisons between groups. Therefore, we performed leg-specific comparisons between the master and young runners. We observed that all the significant group differences were present in both legs, which further confirms that the observed kinematic changes are indeed associated with age and/or sustained running and are not the result of, e.g., limb dominance.

Several limitations to this study should be considered. First, only lower-limb kinematics in the sagittal plane were examined. However, other planes of lower-limb kinematics may also contribute to running economy and performance and can be considered in future investigations of the effect of aging on running biomechanics. Second, kinetic data were not collected and examined in this study, while external kinetics (i.e., ground reaction forces) and joint kinetics (i.e., joint moments) are known to be affected by aging (21, 22, 62, 63) and to be important considerations for running economy and performance (64). Third, a non-exhaustive 30-minute running protocol was used in this study to examine the effect of a typical training run on running biomechanics. Similar protocols have previously been shown to induce alterations in running kinematics (27, 28). Since the runners included in this study were all well-trained, less-trained runners may display more prominent changes in kinematics and further highlight age-related differences in biomechanical responses to sustained running. Fourth, confounding factors, such as physiological and anthropometric differences between male and female runners, were not considered but can influence the observed differences or similarities between age groups. Since this study was a first step to examine the role that the physiological effort at which runners perform plays in biomechanical differences that have been observed between younger and master runners, we encourage future studies to delve into the interactions of other confounding factors (including sex, anthropometrics, etc). Finally, VO_2_ was not measured and monitored during the sustained 30-min run. The assumption that runners maintained a relative physiological effort of 70% of their VO_2_-max could thus not be verified.

## Conclusion

We show that master runners do not have a different step frequency, vertical oscillation of the CoM, or knee range of motion when relative physiological effort was matched with young runners. Speed-matched studies have previously found age-related differences in these characteristics. Those adjustments in master runners may, therefore, not be an inevitable result of aging but rather a strategy to maintain running economy (and consequent performance). However, differences in step/stride length, knee angle at touch-down, and knee stiffness were observed between groups even when the relative physiological effort was matched in all runners. Adjustments in these characteristics are thus more likely to be the result of the aging process. The relative physiological effort at which runners perform should, therefore, be considered when examining age-related adjustments in running biomechanics.

## Data Availability

The raw data supporting the conclusions of this article will be made available by the authors, without undue reservation.
